# Differential proteins among normal cervix cells and cervical cancer cells with HPV-16 infection, through mass spectrometry-based Proteomics (2D-DIGE) in women from Southern México

**DOI:** 10.1186/s12953-016-0099-4

**Published:** 2016-09-05

**Authors:** Idanya Serafín-Higuera, Olga Lilia Garibay-Cerdenares, Berenice Illades-Aguiar, Eugenia Flores-Alfaro, Marco Antonio Jiménez-López, Pavel Sierra-Martínez, Luz del Carmen Alarcón-Romero

**Affiliations:** 1 Laboratorio de Citopatología e Histoquímica, Unidad Académica de Ciencias Químico Biológicas, Universidad Autónoma de Guerrero, Chilpancingo, Guerrero México; 2 Laboratorio de Biomedicina Molecular, Unidad Académica de Ciencias Químico Biológicas, Universidad Autónoma de Guerrero, Chilpancingo, Guerrero México; 3 Instituto Estatal de Cancerología “Dr. Arturo Beltrán Ortega”, Acapulco, Guerrero México; 4 Laboratorio de Investigación en Citopatología e Histoquímica, Unidad Académica de Ciencias Químico Biológicas Universidad Autónoma de Guerrero Avenida Lázaro Cárdenas, Ciudad Universitaria, Chilpancingo, Guerrero C.P. 39090 México

**Keywords:** Cervical cancer, Human Papilloma Virus 16 (HPV-16), Proteomics, 2D DIGE, Mass spectrometry

## Abstract

**Background:**

Cervical cancer (CC) is the fourth most common cancer in women worldwide with an estimated 528,000 new cases in 2012. The same year México had an incidence of 13,960 and a mortality of 4769 cases. There are several diagnosis methods of CC; among the most frequents are the conventional Pap cytology (Pap), colposcopy, and visual inspection with acetic acid (VIA), histopathological examination, tests of imaging and detection of high-risk papilloma virus (HR-HPV) with molecular tests (PCR, hybridization, sequencing). Proteomics is a tool for the detection of new biomarkers that can be associated with clinical stage, histological type, prognosis, and/or response to treatment. In this study we performed a comparative analysis of CC cells with normal cervical cells. The proteomic analysis was carried out with the fluorescent two-dimensional electrophoresis (2D-DIGE) technique to subsequently identify differential protein profiles using Decyder Software, and the selected proteins were identified by Mass Spectrometry (MALDI-TOF).

**Results:**

The proteins that showed an increased expression in cervical cancer in comparison with normal cervix cells were: Mimecan, Actin from aortic smooth muscle and Lumican. While Keratin, type II cytoskeletal 5, Peroxiredoxin-1 and 14-3-3 protein sigma showed a decrease in their protein expression level in cervical cancer in comparison with normal cervix cells.

**Conclusions:**

Thus, this study was successful in identifying biomarker signatures for cervical cancer, and might provide new insights into the mechanism of CC progression.

## Background

Cervical cancer is the fourth most common malignancy and accounts for 10–15 % of cancer-related deaths in women worldwide [1, 2]. Cervical cancer affects approximately six out of 100,000 women and accounts for approximately 275,000 deaths annually in developing countries, which corresponds to 88 % of cases worldwide [2, 3].

Human papillomavirus (HPV) is the main etiological agent of cervical cancer detected in 95 to 100 % of cases [4–6]. Although more than 150 variants of this virus exist, only certain genotypes, such as HPV 16, 18, 33, 45 and 58 are known as high-risk types (HR-HPV); low-risk HPV types (LR-HPV), mainly HPV 6 and 11, seldom cause genital tumors; however, they do cause condylomata acuminata (anogenital warts) [7]. Persistent HPV oncoprotein expression (E6/E7) in HPV infected epithelial basal cells deregulates cell division [8]. Overexpression of these viral genes causes the deregulation of cell proliferation, metabolism, apoptosis, differentiation and genomic instability, all of which may lead to consecutive stages of cervical cancer [9].

Current approaches for the prevention of cervical cancer relies mainly on the cytologic screening, known as the Pap test, often combined with the detection of high-risk human papillomaviruses (HR-HPVs) [10]. Patients with abnormal Paps undergo colposcopy with directed biopsies. If precursor lesions are identified, patients are treated by cryotherapy or loop electrosurgical excision procedure (LEEP). The treatment is effective in the prevention of cervical cancer; however, it is expensive, cumbersome, and dependent on very good infrastructure and well-trained personnel [11]. Nevertheless, the diagnosis may results in a poor outcome, which lies on the lack of valuable objective indicators for determining cervical chronic inflammation, reactive hyperplasia, and benign or malignant lesions [12].

Compared with conventional 2-DE, two-dimensional differential in-gel electrophoresis (2D-DIGE)-based quantitative proteomics has several advantages, such as higher sensitivity, accuracy, and reproducibility, which facilitate spot-to-spot comparisons, precisely because of pre-labeling of protein samples with different fluorescent dyes (Cy3, and Cy5) prior to separation by 2-DE [13]. As a result, samples labeled with different dyes are separated in the same 2D gel; moreover, the same internal standard is used in all gels to avoid inter-gel variation [12, 14]. In the present study, a differential proteomic technique was applied for the comparative analysis of cervical cancer samples infected with HPV 16 and normal cervical tissue.

## Results

**Fig. 1:**
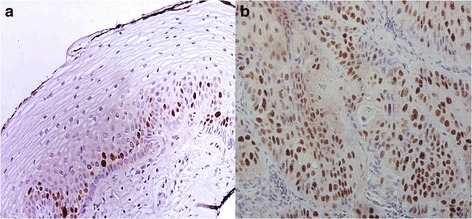
Expression of Ki-67. **a** Without SIL and HPV infection tissue showing nuclear immunostaining was exclusively confined to the parabasal layers of normal epithelium (**b**) SCC and HPV-16 infection tissue showing strong immunostaining of Ki-67 in large pleomorphic nuclei of malignant squamous cells form irregular nests invading the stroma. 40 X Immunohistochemistry

**Fig. 2:**
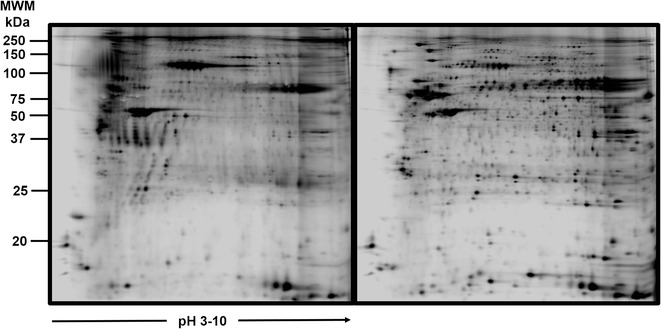
Bidimensional electrophoresis of squamous cell carcinoma HPV-16 pool vs control cervical cells without HPV infection pool. Gel representative of processing samples, normal cervical cells (left), cervical cancer (rigth). More than 2000 proteins were resolved

To carry out a comparative analysis of cervical cancer/HPV-16 and normal samples without HPV, a proteomic analysis was done. Two groups of pooled samples were used. The first one consisted of six samples from women (average age of 50.7 years) diagnosed with HPV-16, by the INNOLIPA assay, and with histopathological staging of squamous cell carcinoma (SCC) of which four cases were stage IIB, one stage IB1 and one case stage IB2. The second group consisted of four pooled samples from women (mean age of 49.8 years) with normal cytology and colposcopy and negative for HPV infection. In Fig. 1 is observed the proliferation index of the cervical tissues by immunohistochemistry. Figure 2 shows the protein profile of each pooled sample, used as a reference pattern, allowing the visualization of more than 2204 spots that were resolved for each 2D gel analyzed. The samples were pooled in two groups and analyzed by triplicated and whose profiles showed technical reproducibility.

**Fig. 3:**
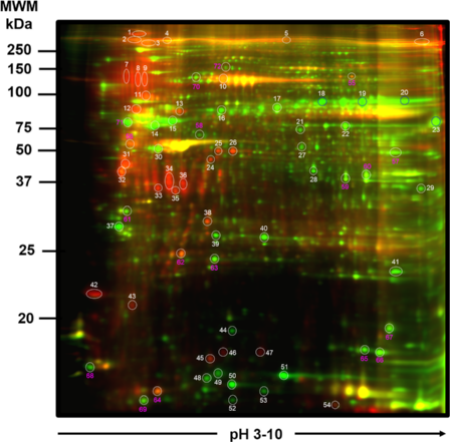
Representative 2D-DIGE proteome map of control cervical cells sample vs cervical cancer sample. (Control cervical cells without HPV 16 infection, stained with Cy3 (green), and a cervical cancer group stained with Cy5 (red), and overlapping of two groups stained with Cy3 and Cy5 (yellow). The samples were processed through PAGE at 10 %

To compare samples, Decyder software (www.gelifesciences.com) was used, which is a platform that allows the qualitative and quantitative evaluation of spots profiles from fluorescent bidimensional electrophoresis gels (DIGE) (Fig. 3).

**Fig. 4:**
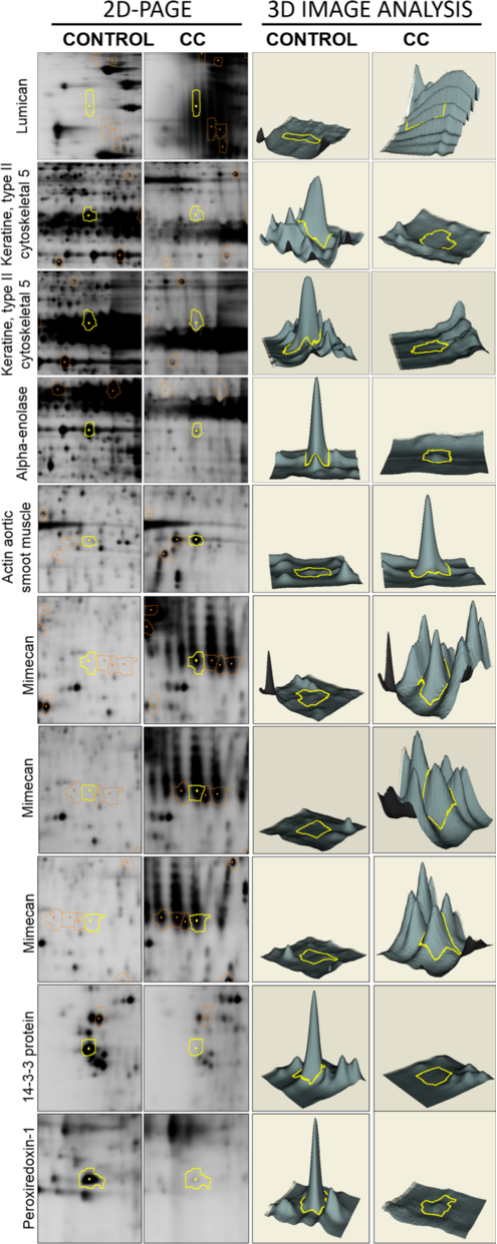
Comparison of protein expression levels among normal cervical cells without HPV infection, and cervical cancer HPV16 infection groups. Decyder software allowed the tridimensional comparisons between study groups

The profile of proteins achieved under these conditions was reproducible among pools, the comparison showed different areas of differential expression (yellow spots). An area with a differential expression pattern among samples was located, from which 129 spots with a diminishing expression and 150 with increased expression +/− 5 times, 53 spots showed a greater differential expression and the identification of the ten spots was achieved by Mass Spectrometry (MALDI-TOF). Figure 4 shows that Mimecan, Actin aortic smooth muscle and Lumican increased their expression in cervical cancer, in comparison with normal cervical cells, while Keratin, type II cytoskeletal 5, Peroxiredoxin-1 and 14-3-3 protein sigma showed a decrease in their protein expression pattern in cervical cancer in comparison with no SIL tissues.

Table 1 shows the identification of proteins and some biochemical characteristics as name, identification code, and chromosomal localization among others.

**Table 1:** Differential expression of proteins in SCC/HPV-16 against control (without SIL and HPV infection)

Protein	Expression index (n+/−)	Accession number	Chromosomal localization	Gene	Peptide coverage (%)	Score
Mimecan	38.85	MIME_HUMAN	9q22	OGN	46	956
Mimecan	31.23	MIME_HUMAN	9q22	OGN	54	820
Actin, aortic smooth muscle	19.14	ACTA_HUMAN	10q23.31	ACTA2	59	311
Mimecan	17.77	MIME_HUMAN	9q22	OGN	54	447
Lumican	16.52	LUM_HUMAN	12q21.33	LUM	40	556
Peroxiredoxin-1	−8.37	PRDX1_HUMAN	1p34.1	PRDX1	79	968
14-3-3 protein sigma	−14.97	1433S_HUMAN	1p36.11	SFN	67	977
Alpha-enolase	−17.38	ENOA_HUMAN	1p36.2	ENO1	71	976
Keratin, type II cytoskeletal 5	−39.7	K2C5_HUMAN	12q13.13	KRT5	52	885
Keratin, type II cytoskeletal 5	−65.34	K2C5_HUMAN	12q13.13	KRT5	52	1 090

Analyzing the participation of the identified proteins in different cellular pathways through Reactome Database (www.reactome.org), the results suggest biosynthesis, metabolism and degradation of keratan sulfate, transcriptional regulation by TP53, metabolism of carbohydrates, intrinsic pathway for apoptosis, detoxification of reactive oxygen species, and cell cycle as the most important.

## Discussion

In 2012 the CC was the second cause of cancer death in Mexican women [15], HPV-16 has been reported in studies carried out by our group, as the more common in populations of women of the southern region of Mexico since 1997 [16, 17].

Multiple genes are involved in the occurrence and development of cancers, and even tough genes carry the genetic information, proteins are the final executors of life events [1]. Due to this, the proteomic profiling offers an option for the selection of differential protein patterns whose expression levels could be associated with progression, prognosis and/or survival.

There have been several experimental approaches for the analysis of differential protein expression in cervical cancer, such as the analysis of tissues [12], cells [1], and even plasma samples [12]. In this study, cells that come from women with cervical cancer and infection with HPV-16 were compared with normal cells of the cervix recovered from individuals without HPV infection, diagnosed through INNOLIPA assay and *in situ* hybridization with tyramide amplification.

Bidimensional electrophoresis is a very useful tool to analyze broad and complex samples, through the wide distribution of protein profiles based on their isoelectric points and molecular weights. The additional use of fluorescence during this technique allows for an increase in sensitivity in 2D DIGE, and the use of special software facilitates the differential analysis. Finally, the use of MALDI-TOF MS, allowed the successful identification of ten differentially expressed proteins among the two different groups.

In the present study, the proteins identified were mimecan (osteoglycine), aortic smooth muscle, Lumican, Peroxiredoxin-1, 14-3-3 protein sigma, Alpha-enolase, Keratin, type II cytoskeletal 5, as differential proteins among cervical cancer and normal cervical cells, whose expression patterns were either increased or diminished. Mimecan (or Osteoglycin, OGN), is a secretory protein that belongs to a family of small leucine rich proteoglycan (SLRPs). It has been found in several cancer cell lines, although its physiological function has not been completely understood [18, 19], being abundant in bone matrix, cartilage cells, and connective tissues; it is also important for collagen fibrillogenesis, cellular growth, differentiation and migration [20] and it has been involved in the pathogenesis of different cancers such as colorectal [21], laryngeal carcinoma [19] and cervical cancer.

The actin cytoskeleton is substantially altered in cancer cells as a result of the changes in the abundance of proteins, among other factors. As a result, cancer cells acquire increased motility and distinctive mechanical properties, which are important for processes such as invasion and metastasis [22]. ACTA2 is a α-smooth muscle actin whose expression is transformation sensitive to growth signals in normal cells [23].

In cases of basal cell carcinoma skin cancer, it has been found with a high expression too, both in the tumor and in the adjacent stroma and it is used as a marker of aggressiveness in pancreatic cancer, because it is presenting more aggressive histological variants [24], suggesting that the increased expression may contribute to the local invasion. It’s role in the biology of the tumor is not completely known, however, it has been hypothesized that increases in the cellular motility result in an increase in the cells capacity of invasion [25].

Lumican is a keratin sulphate belonging to the SLRP family of extracellular matrix (ECM) proteins and is expressed in different forms in several tissues and organs, such as cornea, bone, cartilage, artery, skin, kidney, and lung. It has been found to have a key role both in the organization of the extracellular matrix (ECM) and as an important modulator of biological functions in breast, lung, and pancreas cancer [26, 27], and it has been correlated with lung cancer progression as well [28].

The peroxiredoxins were identified primarily by their ability to protect the protein oxidative damage induced by free radicals, such as reactive oxygen species (ROS) and reactive nitrogen species (RNS), which is currently recognizedas a promotor for cancer development [29]. The peroxirredoxins (Prdxs) are small proteins of sweep (scavening) of H_2_O_2_, that could prevent tumor development since the loss of Prdx1 in mice leads to premature death due to cancer [30].

Keratins are expressed in all types of epithelial cells (simple, stratified, keratinized and cornified), are important protectors of epithelial structural integrity under conditions of stress, but have also been recognized as regulators of other cellular functions, including motility,signaling, growth and protein synthesis [31]. KRT5 Keratin, type II cytoskeletal 5 was found decreased in primary early-stage cervical squamous cell cancer tissue with pelvic lymph node metastasis (PLNM) vs without PLNM using DIGE-based proteomics [32].

## Conclusion

Based on the above results, it was possible to identify differential protein patterns among cases of cervical lesions related to cervical cancer progression with HPV-16 infection in comparison with tissue without lesion and negative for HPV infection from Mexican women. These proteins could be analyzed as potential candidates for biomarkers due to their relationship with their presence in early stages of cervical cancer development, improving the understanding of viral pathogenesis and its role in the development of cervical cancer. Eventually this information could be used in the development of strategies in clinical management, diagnosis and treatment.

## Methods

### Study population

A cross-sectional study was conducted, which included two study groups, the first included a pool of 6 cases of cervical cancer with HPV-16 infection (cervical cancer/HPV-16) of which four cases were stage IIB, one stage IB1 and one case stage IB2 and the second group, which served as a comparison, consisted of a pool of four cases from surgical specimens extracted by fibroids without lesions related to cervical cancer and without HPV infection, and whose previous cytological study showed normal cells with reactive changes to nonspecific inflammatory processes, with normal colposcopy. All cases had antecedents cytological that were reported according to the Bethesda System 2001 and without coninfection by *Trichomonas vaginalis, Candida sp, Actinomyces sp* and *Herpes simplex virus* [33].

The diagnosis and detection of HPV in each sample was performed as follows: three different sections of each biopsy were obtained. The first embedded in paraffin and were cut to a thickness of 3 μm (one stained with hematoxylin-eosin for histopathological diagnosis, another for detecting cell proliferation antigen Ki-67 and finally another slide for detection of DNA detection HPV by *in situ* hybridization with tyramide amplification). The second section was used for detection of HPV DNA by molecular analysis (INNOLiPA, Uniparts Innogenetics) and the third was used for protein extraction. These data allowed the formation of the two comparative groups (Table 2).

**Table 2:** Characteristics of cervical tissues samples of comparatives groups CC/HPV-16 and control without SIL and HPV infection

Tests^a^	Pool CC/HPV-16	Pool without SIL and HPV infection (control)
Background	Cytological^b^ study: Squamous cell carcinoma Colposcopic examination: cervical carcinoma Clinical diagnosis: 4 cases stage IIB, one stage IB1 and 1 case stage IB2	Cytological^b^ study: normal cells with reactive changes to nonspecific inflammatory processes Normal colposcopy
Section 1 (paraffin embedding) - Histopathological diagnosis (H-E) - *in situ* hybridization with tyramide amplification - Immunohistochemistry for Ki-67	Squamous Cell Carcinoma Positive of HR- HPV Positive in large pleomorphic nuclei of malignant squamous cells form irregular nests invading the stroma	Negative Negative Positive in nuclei of parabasal cells in basal layer
Section 2 - INNO-LIPA extra (Genotyping of HVP)	HPV-16	Negative of HPV infection
Section 3 - Extraction of proteins for 2D- DIGE and MALDI-TOF	Increased expression in cervical cancer Mimecan, Actin from aortic smooth muscle and Lumican. Decreased expression: Keratin, type II cytoskeletal 5, Peroxiredoxin-1 and 14-3-3 protein sigma	

^a^Each sample of tissue fragment into three sections and all tests were performed, six samples for group CC / HPV-16 and four samples were used for the control group. For more details see section Methods

^b^ Without coninfection by *Trichomonas vaginalis, Candida sp, Actinomyces sp, Herpes simplex virus* according to the Bethesda 2001 system

The cervical cancer tissues were obtained from the Instituto Estatal de Cancerología “Arturo Beltrán Ortega”, and normal cervix tissues from patients from the Hospital General “Dr. Jorge Soberón Acevedo”. The histopathological diagnosis was carried out by pathologists in each institution independently, according to each clinical record. Both groups contained women with no history of prior local treatment, chronic degenerative diseases, non-smoking and non-alcoholism history, and without coinfections corroborated by colposcopic, cytological and microbiological analyses. The diagnosis was reported in accordance with the system of the International Federation of Gynecology and Obstetrics (FIGO) [34]. Ages in both groups were 37–69 years with a mean age of 50.7 years among cervical cancer patients and 49.8 among patients free-cervical. Women signed an informed consent and their data were analyzed in a confidential manner. This study was approved by the Committee of Bioethics at the Autonomous University of Guerrero, Mexico, according to the ethical guidelines of the Declaration of Helsinki 2008 [35].

### Immunohistochemistry for Ki-67

To evaluate the expression of Ki-67, the monoclonal antibody MIB-1 (Dako, Carpinteria, CA, USA) was used with the Cytoscan HRP/DAB immunohistochemical system of detection (Cell Marque Corporation, Hot Springs, AR, USA [now relocated to Rocklin, CA, USA]). The histological slices was deparaffinized and placed in a solution of immunoDNA Retriever (BioSB, Inc., Santa Barbara, CA, USA). Later, the primary antibody, previously diluted in accordance with the manufacturer’s instructions, was added; the chromogen diaminobenzidine was added; and finally, the specimens were stained with Mayer’s hematoxylin (Merck, USA). The expression in the without SIL tissues was evaluated in accordance to the distribution and localization of the positive reaction within the cells and within the depth of the epithelium. The expression of Ki-67 was considered positive when a brown ochre color was evident in the nucleus of the cells. In SCC is observed in large pleomorphic nuclei of malignant squamous cell form irregular nests invading the stroma and tissue without SIL some cells of the parabasal layers were found (Fig. 1).

### Detection and genotyping of HPV DNA by in situ hybridization with tyramide amplification and INNO-LiPA Extra

In the *in situ* hybridization with a system of tyramide signal amplification (Gen Point Dako Cytomation, Carpinteria, CA, USA), a drop of test reagent (biotinylated viral DNA) with probes for 13 HR-HPV genotypes (16,18,31,33,39,45,51,52,56,58,59 and 68) was added to each slide. The slide were denatured for 10 min and subjected to hybridization for 20 h (Hybridizer Dako, Carpinteria, CA,USA). For HVP-INNO-LIPA extra, the DNA extraction from cervical tissue samples the conventional TRIZOL method (Cat. No.15596018, Invitrogen a part of life Tech. Corp) was used, according to the manufacturer’s instructions. Tissues were tested for the presence of the HPV genotypes by polymerase chain reaction (PCR), using the short PCR fragment 10 (SPF10) primers, a highly sensitive method for HPV DNA detection, according to the method of Pirog et al. [36].

### Protein sample labeling with CyDye

CyDye DIGE fluors (Cy2, Cy3, and Cy5) were used to label the protein extracts following the manufacturer’s protocol (GE Healthcare). The internal standard pool was generated by combining equal amounts of extracts from all samples, labeled with Cy2. Protein extracts from the normal and cervical cancer cells were labeled with Cy3 and Cy5, respectively. The labeling reaction was performed on ice for 30 min in darkness, and was then quenched with 10 mM lysine for 10 min on ice under dark conditions. The labeled samples were then mixed and prepared for the following steps.

### 2-D electrophoresis in polyacrylamide gel electrophoresis (SDS-PAGE)

Total extracts from normal and cancer tissues were processed according to Klose protocol [37]. Briefly, samples were diluted in rehydration buffer containing 8 M urea, 0.5 % (w/v) CHAPS, 10 mM DTT, 0.001 % bromophenol blue, and Bio-Lyte 3–10 Ampholyte (0.2 %) (Bio-Rad, Cat. No.163-1113). The protein mixture was then applied to ReadyStrip™ IPG 7 cm strips, pH 3–10 (linear). Rehydrated strips were isoelectrically focused using a PROTEAN IEF cell System. To perform the second dimension analysis, the strips were processed by 10 % SDS-PAGE. Finally the 2D gels were stained with Coomassie staining. The IPG strips were balanced in a buffer consisting of 1.5 M Tris–HCl pH 8.8, 6 M urea and 30 % v/v glycerol, 2 % p/v SDS and traces of bromophenol blue, being carried out in two steps, using in each 5 ml of balanced solution per strip; in the first one, the strips were incubated in the balanced buffer described above with 1 % w/v of DTT, remaining in this solution for 20 min in agitation at room temperature. In the second step, 2.5 % w/v of iodoacetamide was added to the balanced buffer, repeating the 20 min incubation in agitation at room temperature; once the procedure was completed, the proteins could be picked for mass spectrometry analysis.

### Image analysis

The images were analyzed using the DeCyder™ 2D Differential Analysis Software (DeCyder 2D V8.0) by differential in-gel analysis (DIA) and biological variation analysis (BVA). Protein spots were marked and selected with changes in abundance ratio >1.5-fold, *P* values <0.05 for protein identification. The gels were subjected to Coomassie blue staining for spot visualization and picking.

### Protein identification by MALDI-TOF

Commassie-stained 2D gels were scanned and digital images were compared using the Decyder Software. Each pool was run three times. The electrophoretic entities of interest were excised, alkylated, reduced, and digested up to obtain a peptide mass fingerprint. Peak lists of the tryptic peptide masses were generated using FlexAnalysis1.2vSD1 Patch 2 (Bruker Daltonics). The search engine MASCOT server 2.0 was used to compare the fingerprints against human taxonomy with the following parameters: one missed cleavage allowed, carbamidomethyl cysteine as the fixed modification and oxidation of methionine as the variable modification. Proteins with scores greater than 50 and a *p* < 0.05 were accepted.

## Abbreviations

ECM: Extracellular matrix
FIGO: International Federation of Gynecology and Obstetrics
HR-HPV: High-risk papilloma virus
MALDI-TOF: Matrix-Assisted Laser Desorption/Ionization-Time of Flight
OGN: Osteoglycin
Prdxs: Peroxirredoxins
ROS: Reactive oxygen species
SCC: Squamous cell carcinoma
SIL: Squamous Intraepithelial Lesion
SLRP: Small leucine rich proteoglycan
